# BOF steel slag as a low-cost sorbent for vanadium (V) removal from soil washing effluent

**DOI:** 10.1038/s41598-017-11682-3

**Published:** 2017-09-11

**Authors:** Yuchen Gao, Jianguo Jiang, Sicong Tian, Kaimin Li, Feng Yan, Nuo Liu, Meng Yang, Xuejing Chen

**Affiliations:** 1 School of Environment, Tsinghua University, Beijing, 100084 China; 2 Key Laboratory for Solid Waste Management and Environment Safety, Ministry of Education of China, Beijing, 100084 China; 3 Collaborative Innovation Center for Regional Environmental Quality, Tsinghua University, Beijing, 100084 China

## Abstract

Soil washing is an effective remediation method to remove heavy metals from contaminated soil. However, it produces wastewater that contains large amounts of heavy metals, which lead to serious pollution. This study investigated the removal of vanadium (V) from synthetic soil washing effluent using BOF steel slag. The effects of particle size, slag dosage, initial pH, and initial vanadium concentration on removal behavior were studied. Adsorption kinetics and isotherms were also analyzed. The results showed that the vanadium removal efficiency increased as the steel slag particle size decreased and as the amount of slag increased. The initial pH and vanadium concentration did not play key roles. At the optimum particle size (<0.15 mm) and dosage (50 g/L), the removal rate reached 97.1% when treating 100 mg/L of vanadium. The influence of the washing reagent residue was studied to simulate real conditions. Citric acid, tartaric acid, and Na_2_EDTA all decreased the removal rate. While oxalic acid did not have negative effects on vanadium removal at concentrations of 0.05–0.2 mol/L, which was proved by experiments using real washing effluents. Considering both soil washing effect and effluent treatment, oxalic acid of 0.2 mol/L is recommended as soil washing reagent.

## Introduction

The soil in many regions of the world is polluted by heavy metals. With the rapid development of the vanadium industry, vanadium pollution in soil has become a serious problem in countries such as Russia, China, and South Africa^1^. Vanadium can damage plant growth, reduce agricultural production^2^, and harm human health via the food chain. Soil washing is an effective remediation method that can efficiently remove heavy metals such as chromium (Cr)^3, 4^, lead (Pb)^5^, copper (Cu)^6^, zinc (Zn)^5–7^, arsenic (As)^8^, and cadmium (Cd)^6, 8^ from soil. However, a large volume of washing effluent containing heavy metals is discharged in this process. Jung *et al*.^9^ used micellar-enhanced ultrafiltration to remove over 92% of the Cu, Zn, Pb, and Cd from soil washing effluent. Sequential photocatalytic processes, or combined techniques of physical adsorption and sacrificial photocatalysis are used to remove Cu, Zn, iron (Fe), and manganese (Mn) from soil washing effluents^10^. Few studies have examined the disposal of soil washing effluent containing vanadium, although industrial wastewater containing vanadium has been decontaminated using methods such as precipitation^11, 12^, adsorption^13, 14^, ion exchange^15^, electrolysis^16^, solvent extraction^17^, and biological treatment^18^. Precipitation is a commonly used and reliable method, but it requires the addition of large amounts of chemicals and subsequent treatment is needed. Adsorption is an effective way to remove heavy metals, but commonly used adsorbents, such as activated carbon, are relatively expensive. Ion exchange and solvent extraction require a large investment in equipment, which limits their use. Electrolysis and biological methods are still being evaluated in the laboratory and need further research before their application. Therefore, a cost-effective, environmentally benign technology is required for disposing of vanadium-contaminated soil washing wastewater.

Steel slag is a common byproduct of the steel-making industry, and large amounts are produced annually. Steel slag is a complex mixture of oxides, such as CaO, MgO, and SiO_2_, which can undergo hydration reactions with H_2_O, increasing the alkalinity, adsorption capacity, and chemical precipitation ability of solutions^19^. Traditionally, steel slag is used mainly in road and hydraulic construction and performs favorably due to its high strength and bulk density. Such applications are used widely in China^20^, Germany^21^, and Japan. In addition, steel slag can serve as a sintering material^22^ due to its high CaO content and can be used to produce cement and concrete by virtue of its cementitious properties^23^. However, these traditional applications cannot satisfy the demand for steel slag disposal. Some applications, such as road construction, may also have safety risks due to the potential expansion of steel slag on reaction with water. Therefore, many researchers have attempted to develop new uses for steel slag. Sun *et al*.^24^ used steel slag as a CO_2_ sorbent, achieving a maximum CO_2_ uptake of 211 kgCO_2_/ton steel slag. Tian *et al*.^25^ converted steel slag into CaO-based CO_2_ sorbents by structure-reforming, with a maximum CO_2_ uptake of 0.50 g(CO_2_)g(sorbent)^−1^. Miguel *et al*.^26^ investigated the adsorption of H_2_S gas and achieved an adsorption capacity of 180 mg H_2_S /g slag. Zhang *et al*.^27^ found that steel slag could decompose the greenhouse gas SF_6_ via a redox reaction.

Many recent studies have focused on wastewater treatment using steel slag. Claveau-Mallet *et al*.^28^ used steel slag filters to remove phosphorus from water and developed a phosphorus-retention mechanisms model. Later, they reported the forward kinetic constants for the model to predict the longevity of the filters^29^. Asaoka *et al*.^30^ and Kim *et al*.^31^ focused on H_2_S removal from aqueous solutions using steel slag, with a maximum adsorption of 37.5 mg S/g. Moreover, steel slag was able to remove several heavy metals from wastewater. Liu *et al*.^32^ studied the kinetics and isotherms of Pb^2+^ adsorption on steel slag and achieved a maximum removal efficiency of 99%. Kim *et al*.^33^ investigated the removal characteristics of Cu using steel slag and found that the main mechanisms were adsorption and precipitation. Xue *et al*.^19^ also studied Cu removal and achieved a removal rate of 99.9%. Oh *et al*.^34^ found that the formation of amorphous CaCO_3_ played an important role in As(III) and As(V) removal, with an efficiency exceeding 95%. Ochola *et al*.^35^ found that modified steel slag could remove Cr(VI) effectively via a reduction mechanism. Duan *et al*.^36^ tested the removal effect of Cd(II) of unmodified and modified steel slag, concluding that modification remarkably increased the adsorption capacity of steel slag, with a maximum removal rate of 99.1%. Repo *et al*.^37^ investigated the efficiency and mechanism of Co, Ni, Cd, and Pb removal from aqueous solutions containing washing agents using steel slag. They showed a clear dependency of metal speciation and removal behavior and suggested that precipitation and adsorption were the main mechanisms.

This study focused on the efficiency of vanadium removal by basic oxygen furnace (BOF) steel slag under different conditions. Batch experiments were conducted to investigate the influence of steel slag particle size, slag dosage, initial pH, and initial vanadium concentration. Several models were applied to study the kinetics and isotherms of vanadium adsorption on steel slag. The effects of four common soil washing reagents (citric acid, tartaric acid, oxalic acid, and Na_2_EDTA) residue were also studied to simulate real conditions, and provide a reference for choosing the washing reagents for soil washing procedures.

## Materials and Methods

### Materials and analysis

**Figure 1:**
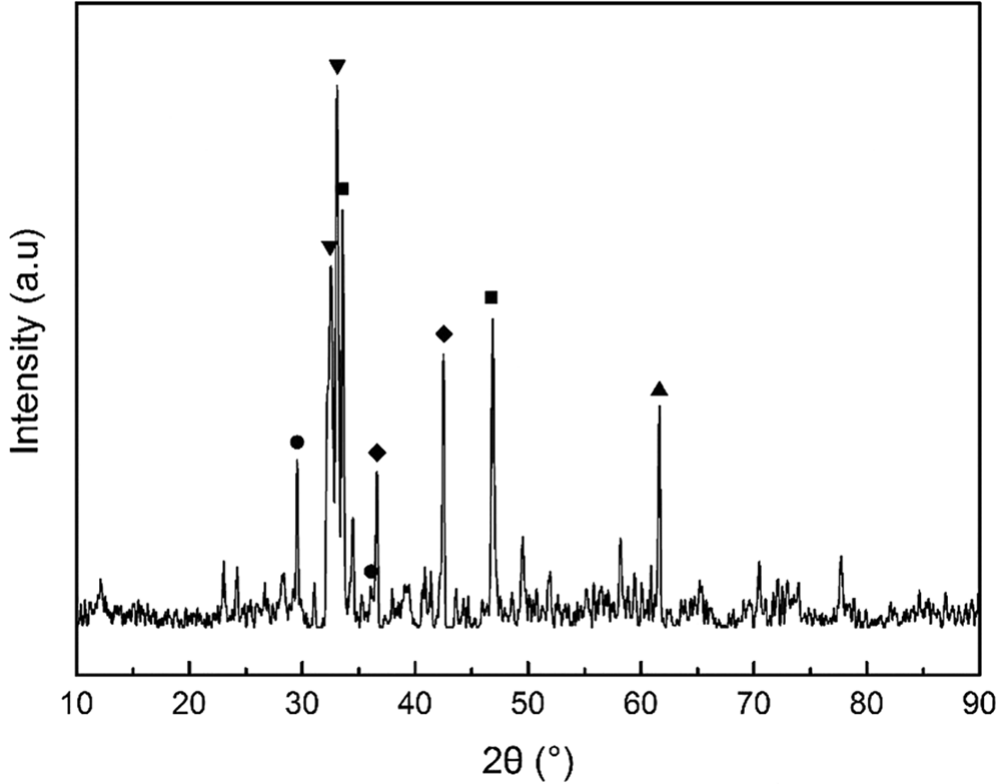
X-ray diffraction (XRD) pattern of the steel slag: (▼) Ca_2_SiO_4_, (■) Ca_2_Fe_2_O_5_, (●) CaSi_2_O_5_, (▲) RO phase-MgO·xMnO, (◆) RO phase-MgO·xFeO.

The steel slag used in this study was obtained from Baosteel, Shanghai, China. The slag was dried at 105 ± 0.5 °C overnight. Before use, the as-prepared sample was crushed and sieved into four different particle size ranges: < 0.15, 0.15–0.5, 0.5–0.9, and 0.9–2 mm. Table 1 lists the chemical composition and physical properties of each sample. Figure 1 shows the x-ray diffraction (XRD) pattern of the steel slag sample.

**Table 1:** The chemical composition (mass %) and physical properties of the BOF steel slag.

Particle size	<0.15 mm	0.15–0.5 mm	0.5–0.9 mm	0.9–2 mm
Fe_2_O_3_ (%)	40.87	43.17	42.70	42.62
CaO (%)	33.66	32.25	32.86	32.90
SiO_2_ (%)	12.54	11.35	11.75	11.93
MgO (%)	6.06	6.51	5.93	5.82
MnO (%)	2.60	2.66	2.65	2.56
P_2_O_5_ (%)	1.34	1.25	1.30	1.34
Al_2_O_3_ (%)	1.09	1.10	1.01	1.01
TiO_2_ (%)	0.82	0.85	0.82	0.81
Others (%)	1.02	0.86	0.98	1.01
BET (m^2^/g)	2.5	0.6	0.6	0.5
Pore volume (cm^3^/g)	0.008	0.002	0.002	0.002
pH values	12.47	12.16	11.86	11.67

The vanadium-bearing solutions for the experiments in this study were prepared by dissolving NaVO_3_ (Aladdin Bio-Chem Technology, Shanghai, China. AR, 99.0%) in deionized water according to the concentration requirement. The solution pH was adjusted with 0.5 M HNO_3_ and 0.5 M NaOH when necessary. The washing reagent residues were simulated by dissolving solid citric acid, tartaric acid (China National Pharmaceutical Group Corporation, Beijing, China. AR, 99.5%), oxalic acid (Beijing Modern Oriental, Beijing, China. AR, 99.5%), or Na_2_EDTA (China National Pharmaceutical Group Corporation, Beijing, China. AR, 99.0%) in the V-bearing solutions.

The chemical compositions of the samples were determined by x-ray fluorescence spectrometry (XRF-1800, Shimadzu, Kyoto, Japan). XRD patterns of the samples were obtained using a D8 Advance x-ray diffractometer (Siemens, Munich, Germany) with diffraction angles (2θ) ranging from 10° to 90°. A shaking table (SHA-BA, Jintan, China) was used for the batch experiments. The specific surface area and porosity of the samples were determined by the Brunauer, Emmett, and Teller (BET) method using the nitrogen sorption isotherms at 77 K, with a surface area and porosity analyzer (ASAP 2020 HD88, Micromeritics, Norcross, GA, USA). The pH of the solutions was determined using a pH meter (PHSJ-3F, INESA; Shanghai, China). The V concentration in the solutions was measured using inductively coupled plasma atomic emission spectrometry (ICP-AES, Thermo Fisher Scientific, Waltham, MA, USA).

The pH value of steel slag samples was tested according to *Solid waste-Glass electrode test-Method of corrosivity* (GB/T15555.12-1995). Put 100 g (dry base) sorbent into a 2 L high pressure polyethylene bottle, then add 1 L deionized water into the bottle. Fix the bottle on the oscillator and keep the oscillation of 110 ± 10r/min for 8 h. After oscillation, keep it standing for 16 h. Then separate the liquid and solid using 0.45 μm filter and measure the pH of liquid with the pH meter.

### Batch experiment procedures

Batch experiments were conducted on a shaking table to study the vanadium removal efficiency, with oscillation at 200 rpm. A NaVO_3_ solution was prepared to serve as synthetic soil washing effluent. The initial vanadium concentrations and concentration at time t were measured to calculate the removal efficiency using Eq. 1. The adsorption capacity was calculated by Eq. 2. All liquid samples were filtered through a 0.45-μm membrane before measuring the concentration.

1 $$\eta =\frac{{C}_{0}-{C}_{t}}{{C}_{0}}\times 100 \% $$

2 $${Q}_{t}=\frac{({C}_{0}-{C}_{t})V}{m}$$

where C_0_ is the initial concentration of vanadium in the solutions; C_t_ is the vanadium concentration at time t; V is the volume of solution; m is the mass of steel slag; ${\rm{\eta }}$ is the removal efficiency and Q_t_ is the adsorption capacity at time t. The methods used to examine the effects of the different factors are described below.

### Effect of particle size

At room temperature (25 °C), 5.0 g of steel slag was added to 100 mL of NaVO_3_ solution with a vanadium concentration of 100 mg/L. The initial pH of each solution was adjusted to 6. The sample particle sizes were <0.15, 0.15–0.5, 0.5–0.9, and 0.9–2 mm. For each particle size, 0.5 mL samples were taken from the reaction at regular intervals from 0 to 24 h, and diluted to 10 mL in a 10-mL volumetric flask. The vanadium concentrations of the solutions after dilution were measured to calculate the removal efficiency at different time points.

### Effect of the initial pH

At room temperature, 5.0 g of steel slag was added to 100 mL of NaVO_3_ solution with a vanadium concentration of 100 mg/L. The initial pH of the solution was adjusted to 1, 3, 5, 7, 9, or 11. The steel slag particles used were <0.15 mm. The pH was determined when the steel slag was first mixed with the solution and after 18 h. After the 18 h reaction, the vanadium concentration was measured. Each experiment was performed in duplicate.

### Effect of dosage and initial V concentration

At room temperature, 5.0 g of steel slag was added to 100 mg/L of NaVO_3_ solution and oscillated for 18 h. The vanadium concentrations tested were 5, 10, 20, 40, 60, and 100 mg/L. The initial pH of the solution was adjusted to 6. The slag particle size used was <0.15 mm. The same experiment was repeated for slag amounts of 0.5, 1.0, and 2.5 g. Each experiment was performed in duplicate.

### Effect of washing reagent residue

At room temperature, the NaVO_3_ and washing reagent were dissolved and diluted to give solutions with a vanadium concentration of 100 mg/L. The solutions were shaken for 4 h at 250 rpm in order to simulate the soil washing process. Then, 5.0 g of the steel slag was added to 100 mL of NaVO_3_ solution and reacted for 18 h, as described above. The concentrations of each washing reagent (citric acid, tartaric acid, Na_2_EDTA, and oxalic acid) in the solutions were 0.05, 0.1, 0.2, 0.3, and 0.4 mol/L. Each experiment was performed in duplicate.

## Results and Discussion

### Effect of particle size

**Figure 2:**
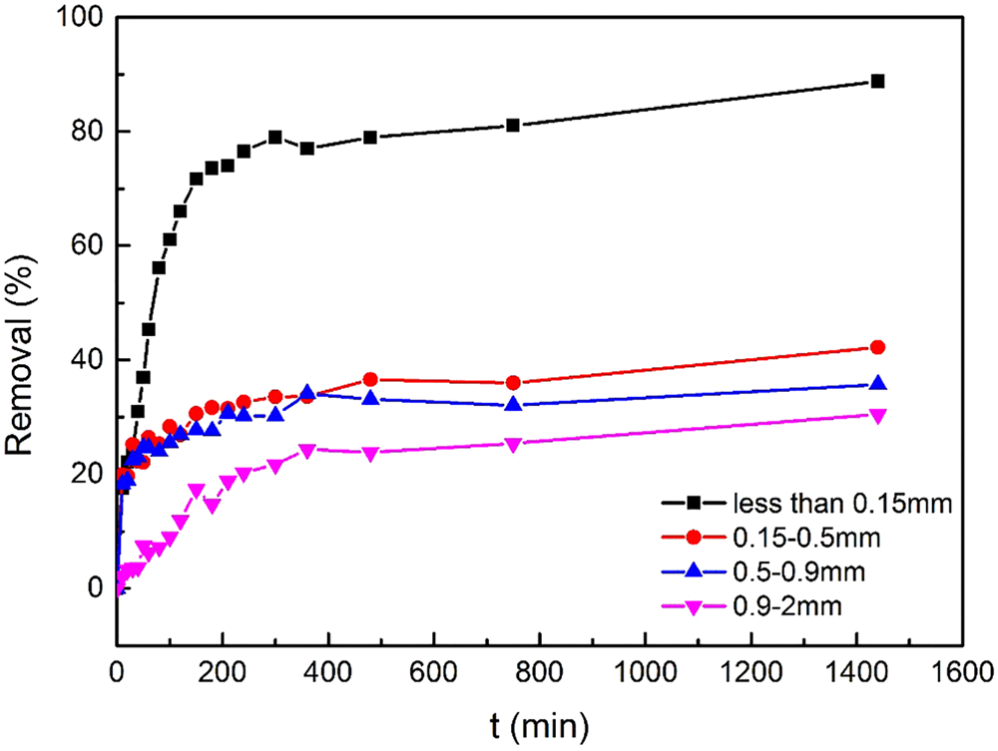
Effect of steel slag particle size on vanadium removal (at room temperature, 200 rpm, initial pH 6, steel slag 5.0 g, initial vanadium concentration 100 mg/L).

Figure 2 shows the change in vanadium removal behavior with time for different slag particle sizes. When the initial vanadium concentration was 100 mg/L and there was 5.0 g of steel slag in each 100 mL of solution, for each condition, the removal efficiency increased rapidly during the initial stage. After about 100 minutes, the increase became slowly and reached a maximum after 24 h. For the steel slag particle sizes 0.15-2 mm, the trends of removal efficiency were similar, with the maximum efficiency between 30.5% and 42.2%. In contrast, steel slag particles <0.15 mm had an outstanding effect on vanadium removal. During the first 200 minutes, the removal efficiency increased rapidly. At the end of a 24-h reaction, the removal efficiency reached 89%, which was more than 2 times of that of other particle sizes. For the steel slag used in our research with particle size all less than 2 mm, it has been reported that intra-particle diffusion is the control step of adsorption and it is fairly related to particle size^32^. Because of the good adsorption behavior, steel slag with particle size less than 0.15 mm was used for the other experiments in this study.

### Effect of the initial pH

**Figure 3:**
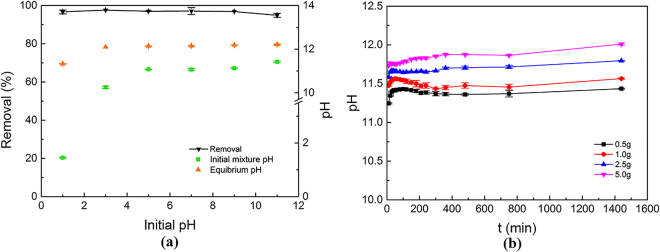
(**a**) Effect of initial pH on vanadium removal and solution pH change (at room temperature, 200 rpm, steel slag 5.0 g, initial vanadium concentration 100 mg/L, 18-hours reaction); (**b**) solution pH change with reaction time under different dosages.

This study focused on the initial pH of the vanadium bearing solutions, because many factors affect the pH of real soil washing effluents, such as the types of the soil and washing reagents. And pH could affect the species of vanadium in solutions (shown in Fig. S5). Figure 3(a) shows that the removal efficiencies were all around 96% when the initial pH of the solution ranged from 1 to 11. This indicates that steel slag with particles <0.15 mm had a good removal behavior as shown in Fig. 2, whereas the initial pH value did not affect the removal percentage. This result is different with some researches using steel slag to remove metal ions like Cd (II) and Cu (II), where initial pH has a major effect on contaminants removal rate^33, 36^. It demonstrates the possible differences of removal mechanisms. Precipitation, which is due to the reactions between metal ions and OH^−^, plays a significant role in the removal of metal ions Cd (II) and Cu (II), while vanadium exists in solutions as anions and could not form precipitate with OH^−^. The wide range of solution pH over which removal was effective broadens the utility of steel slag as an adsorbent, suggesting that steel slag can be applied widely to remove vanadium from different soil washing effluents.

As for solution pH change, the pH measured immediately after the solution and steel slag came into contact (initial mixture pH) and the equilibrium pH after the 18-h reaction were both higher than the initial pH of the solution. When the initial pH of the solution was 1, the initial mixture pH rose only to 1.45, because the initial acidity was too strong to be neutralized within a short period of time during mixture. For other initial pH values, the initial mixture pH exceeded 10. At the end of an 18-h reaction, the equilibrium pH for each condition was approximately 12. Fig. 3(b) clearly shows the process of solution pH change during kinetic tests under different slag dosages, with initial pH being 6. 10 minutes after the reaction began, the solution pH values under different slag dosages have all quickly risen to above 11. At the end of the kinetic test, the pH increased to 11.44, 11.57, 11.80 and 12.01 for dosage 0.5 g, 1.0 g, 2.5 g and 5.0 g, respectively. These high pH values are due to the strong alkalinity released by the steel slag. It has been reported that steel slag increases the solution pH by releasing Ca^2+^ ^37^ and performing hydration reaction with H_2_O to release OH^−^^36, 38, 39^. For each given time t, the pH value increased as steel slag dosage increased from 0.5 g to 5.0 g. This suggests that with the increase of dosage, more Ca^2+^ in slag was released, and thus improved the removal efficiency.

### Adsorption isotherms and kinetics

**Figure 4:**
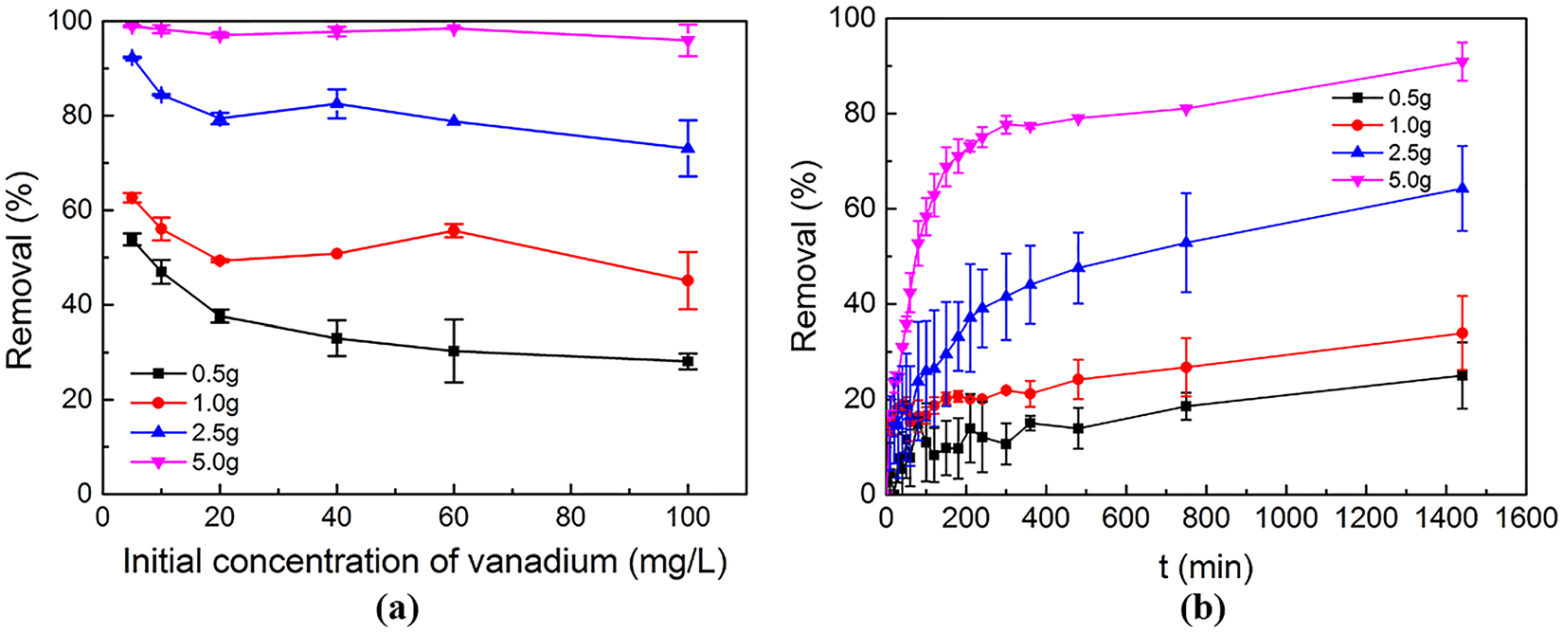
(**a**) Removal efficiency of different slag dosages and initial vanadium concentrations (at room temperature, 200 rpm, initial pH 6, 18-hours reaction); (**b**) kinetic tests of different slag dosages (initial vanadium concentration 100 mg/L).

Figure 4(a) demonstrates the removal efficiencies under different initial vanadium concentration and different dosages of steel slag. For a certain initial concentration, the removal efficiency increased as dosage increased from 0.5 g to 5.0 g. It is obvious that more adsorbents would bring more adsorption amount, when initial vanadium concentration and solution volume are the same. Similar effect of steel slag dosage was also reported in the study of Cd (II) removal by steel slag^36^ and vanadium removal by different iron sorbents^40^. On the other hand, adsorption capacity (Q) calculated via Eq. 2 showed a decrease trend with the increase of dosage. Taking initial vanadium 100 mg/L as an example, the adsorption capacity was 5.6, 4.5, 2.9 and 1.9 mg/g for dosage 0.5, 1.0, 2.5 and 5.0 g, respectively. This could be explained with mass balance:

3 $${{\rm{C}}}_{{\rm{e}}}={{\rm{C}}}_{{\rm{0}}}\,\mbox{--}\,{{\rm{C}}}_{{\rm{a}}}$$

4 $${{\rm{C}}}_{{\rm{a}}}={{\rm{D}}}_{{\rm{a}}}{\cdot Q}_{{\rm{e}}}$$

Where C_e_, C_0_, and C_a_ are the equilibrium, initial and adsorbed concentrations; D_a_ is the adsorbent dosage and Q_e_ is the adsorption capacity of the adsorbent. For a given C_0_, Q_e_ would be lower as D_a_ is larger, which means lower dosages render higher adsorption capacities. This indicates that when slag dosage is excessive, some adsorption sites of steel slag were still unsaturated during adsorption^36^.

To better describe the adsorption characteristics, the results in Fig. 4(a) were used for analysis of adsorption isotherms. The adsorption isotherms were analyzed by Langmuir^41^ and Freundlich^42^ isotherm models. The integrating equations of the two models are as following:

5 $$\frac{{C}_{e}}{{Q}_{e}}=\frac{1}{{k}_{L}{Q}_{m}}+\frac{{C}_{e}}{{Q}_{m}}$$

6 $${Q}_{e}={k}_{F}{{C}_{e}}^{1/n}$$

where Q_e_ is equilibrium adsorption capacity of steel slag (mg/g); Q_m_ is the maximum adsorption capacity of steel slag (mg/g); C_e_ is the equilibrium concentration of vanadium in aqueous solution (mg/L); k_L_ is the Langmuir adsorption constant (L/mg); k_F_ (L/g) and n are the Freundlich equilibrium constants.

The plots of 1/Q_e_ against 1/C_e_ and lg Q_e_ against lg C_e_ were used for parameters calculation of Langmuir and Freundlich isotherms, respectively (see Fig. S1 for linear fit). The isotherms of vanadium adsorption at room temperature were expressed as Eqs 7 and 8, and the isotherm parameters are listed in Table 2. The R^2^ values of the two models are both larger than 0.9, suggesting that the two isotherm models could both fit the adsorption experiments well. This indicates that the adsorption of vanadium on steel slag could be described as monolayer adsorption and it takes place at specific homogeneous sites of steel slag. To get a broader view of the adsorption capacity of steel slag, the isotherm parameters in other studies concerning different pollutants are also listed in Table 2. In some of these studies, steel slag was modified by mixing with aluminum hydroxide^36^ or chitosan^37^, which lead to a better adsorption capacity. The raw steel slag used in our study is cheaper than modified ones and also showed a good adsorption capacity Q_m_ of 5.446 mg/g.

7 $$\frac{1}{{Q}_{e}}=\frac{3.85}{{C}_{e}}+0.18$$

8 $$\mathrm{lg}\,{Q}_{e}=0.66\,\mathrm{lg}\,{C}_{e}-0.47$$

**Table 2:** Langmuir and Freundlich isotherm parameters for different pollutants removal by steel slag at room temperature.

Pollutants	Langmuir isotherm			Freundlich isotherm		
	Q_m_ (mg/g)	k_L_ (L/mg)	R^2^	k_F_ (L/g)	n	R^2^
Cd(II)^36^	10.16	9.196	0.9957	4.73	3.58	0.7863
P^45^	8.560	0.575	0.999	4.973	7.63	0.660
Pb(II)^32^	0.667	0.221	0.99	0.015	4	0.85
Co(II)^37^	12.38	8.262	—	4.259	5.456	—
Ni(II)^37^	15.14	7.085	—	5.231	1.078	—
V (this work)	5.446	0.0477	0.978	0.337	1.52	0.997

Figure 4(b) shows the change of removal efficiency with time for different amounts of steel slag, where the vanadium initial concentration was 100 mg/L. During the first 200 minutes of the reaction, the removal efficiency increased fast, and the use of 5.0 g of slag already produced the best vanadium removal behavior, with efficiency reaching nearly 80%. For the other conditions, it remained within 40%. At the end of the reaction, the removal efficiencies became 25.0%, 34.0%, 64.3% and 90.9% for dosages 0.5 g, 1.0 g, 2.5 g and 5.0 g, respectively. This indicates that removal efficiency increased with the increasing of steel slag dosage. 5.0 g (50 g/L) is the optimum dosage for vanadium removal from solutions.

The adsorption kinetics were analyzed by Weber-Morris model, which is a typical intra-particle diffusion model^44^, using the experiment data in Fig. 4(b). Several researches have used this model to describe the kinetics of heavy metal adsorption by steel slag^33, 36, 37^. The integrating equations of this model is as following:

9 $${Q}_{t}={k}_{\mathrm{int}}{t}^{1/2}+{\rm{C}}$$

where Q_t_ is the adsorption capacities of steel slag (mg/g) at reaction time t (min); k_int_ is the rate constant; C is the intercept which is related to boundary layer thickness. Plots of Q_t_ against t^1/2^ (see Fig. S2 for linear fit) was used for intra-particle diffusion model. The calculation of kinetic parameters was based on these plots, which were shown in Table 3. The pseudo-first-order model and pseudo-second-order model are also used to analyze the kinetic tests as a reference. And the pseudo-second-order model fits well for the adsorption with R^2^ values larger than 0.9 (See Figs S3, S4 and Table S1 for linear fit and parameters), where the kinetic constant showed a trend of increase with the increase of slag dosage.

**Table 3:** Kinetic parameters for vanadium removal by steel slag.

Dosage(g)	Intra-particle diffusion		
	k_int_ (mg/g min^−1/2^)	R^2^	C
0.5	0.126	0.790	0.662
1.0	0.067	0.766	1.198
2.5	0.074	0.945	0.332
5.0	0.053	0.740	0.119

As mentioned above, steel slag could release Ca^2+^ when mixed with aqueous solutions and increase the pH in the system. While vanadium (V) exists in aqueous solutions as the form of anions, which could react with Ca^2+^ and produce precipitate. Based on the experiment results, the probable main mechanism of precipitation-adsorption was suggested. This mechanism is composed of three reaction process: (i) Some contents of the BOF steel slag dissolve in solutions and release Ca^2+^, (ii) Ca^2+^ and vanadium form precipitant during the reaction in equation (7), (iii) the precipitant be adsorbed onto the steel slag surface. Similar mechanisms have been proposed in the research of phosphate removal using steel slag^38^.

10 $${{\rm{Ca}}}^{2+}+{{{\rm{VO}}}_{3}}^{-}\to {\rm{Ca}}{({{\rm{VO}}}_{3})}_{2}$$

### Effect of soil washing reagents on removal behavior

Citric acid, tartaric acid, Na_2_EDTA, and oxalic acid are four commonly used soil washing reagents which exhibit high removal efficiency of extractable state of vanadium in soil^43^. During soil washing procedures, the washing reagents prevalently remain in the washing effluent and change the properties of the aqueous system. Therefore, it is necessary to analyze the effect of the washing reagent residue on vanadium removal. The concentration of the washing reagents was varied from 0.05 to 0.4 mol/L, to simulate real washing effluent. The steel slag dosage was 5.0 g and the initial vanadium concentration was 100 mg/L, i.e., the same conditions as in the section *Effect of the initial pH*.

**Figure 5:**
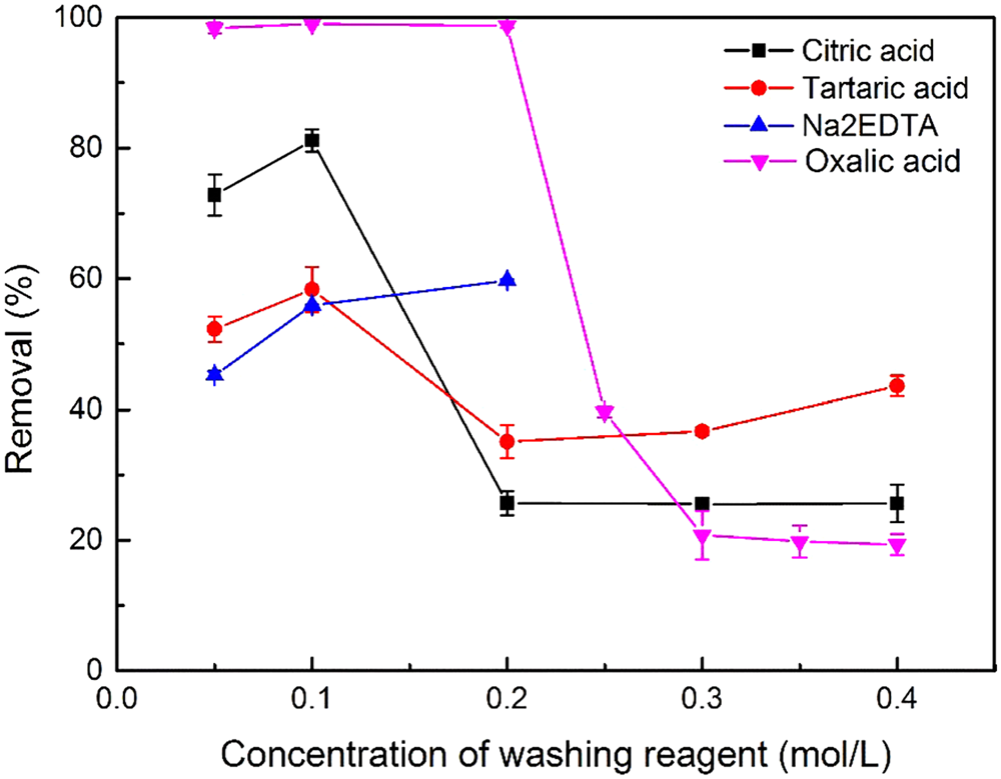
Effects of different washing reagents on vanadium removal (at room temperature, 200 rpm, steel slag 5.0 g, initial vanadium concentration 100 mg/L, 18-hours reaction).

When no washing reagents were added, the removal efficiency reached 97% (see Fig. 3a) and adsorption capacity reached 1.94 mg/g. Figure 5 shows the vanadium removal efficiency when different washing reagents were added to the reaction system. For tartaric acid and Na_2_EDTA, the removal efficiency dropped markedly compared with the result in Fig. 3(a). When the concentrations of tartaric acid were 0.05, 0.1, 0.2, 0.3 and 0.4 mol/L, the removal efficiency decreased from 97% in Fig. 3(a) to 52.3%, 58.3%, 35.1%, 36.7% and 43.6%, respectively, with adsorption capacity lower than 1.2 mg/g. When the concentrations of Na_2_EDTA were 0.05, 0.1 and 0.2 mol/L, the removal efficiency decreased to 45.2%, 55.9%, and 59.7%, respectively. Considering the solubility limit, Na_2_EDTA concentrations higher than 0.2 mol/L were not analyzed. For citric acid, the influence of the washing reagent concentration was more apparent. For concentrations of 0.05 and 0.1 mol/L, the removal efficiency decreased to 72.8% and 81.2%, which is still higher than that of tartaric acid and Na_2_EDTA. However, when the concentration exceeded 0.2 mol/L, the efficiency fell to 25%. The results are in accordance with the reported conclusion that these washing reagents may remobilize metals from sediments and decrease the removal efficiency in water treatment^37^. They act as chelating agents in solutions and have a strong ability to combine with vanadium. This may make it difficult for steel slag to adsorb and react with vanadium in solution, thus brought an unavoidable decrease in vanadium removal efficiency.

It deserves attention that oxalic acid had a different effect on the adsorption reaction. When the oxalic acid concentration increased to 0.25 mol/L, the removal efficiency was as low as 39.6% and became lower as 20% when the acid concentration got higher, similar to other three types of reagents. However, when the oxalic acid concentration was between 0.05-0.2 mol/L, the removal efficiency maintained at a high level of 98%. In comparison with the other washing reagents, it is very favorable that oxalic acid with concentration not higher than 0.2 mol/L had little impact on the vanadium removal efficiency.

As oxalic acid showed an obvious advantage, it was chosen for the study of real washing effluents. 1 L of oxalic acid of (1) 0.1 mol/L and (2) 0.2 mol/L were used to wash 100 g of soil for 4 h respectively with a flip oscillator (28r/min). After that, washing effluents were obtained via centrifugation. 100 ml of the washing effluents of (1) and (2) were treated with 5 g steel slag (<0.15 mm) respectively, at room temperature, 200 rpm, 18 h, which was conducted in duplicate. The metal compositions of washing effluents and the effluents treated by steel slag were listed in Table 4. The vanadium concentrations in real washing effluents (1) and (2) were around 112 mg/L. After treated with steel slag, the vanadium concentrations decreased to 1.90 mg/L and 6.62 mg/L respectively, with removal efficiencies reaching 98.3% and 94.1%. This is in accordance with the results of simulative washing effluents, which proved that oxalic acid with concentration not higher than 0.2 mol/L had little impact on the vanadium removal efficiency. On the other hand, from the perspective of soil remediation, it was reported that oxalic acid of 0.2 mol/L exhibited a vanadium (extractable state) removal rate of nearly 100% in soil washing, and this rate kept stable when oxalic acid concentration exceeded 0.2 mol/L^43^. Therefore, considering both the soil washing behavior and the treatment of washing effluents, oxalic acid of 0.2 mol/L is recommended for washing vanadium contaminated soil. Moreover, from Table 4, the concentrations of other metals in washing effluents also decreased after treated with steel slag, indicating that steel slag could not only remove vanadium from washing effluents but also help remove other heavy metals.

**Table 4:** Metal concentrations (mg/L) in real washing effluents and effluents after treated.

	Cd	Cu	Mn	Pb	Zn	V
Washing effluent (1)	0.04	13.05	4.74	0.01	2.92	**112.10**
Washing effluent (2)	0.05	12.97	4.82	0.02	2.89	**112.30**
Effluent (1) after treated	0	0.35	0.37	0	0	**1.90**
Effluent (2) after treated	0	0.09	0	0	0	**6.62**

## Conclusions

Steel slag was an effective material for removing vanadium (V) from aqueous solution. As the steel slag particle size decreased from 0.9–2 mm to less than 0.15 mm, the removal efficiency increased notably. The removal rate was around 96% for initial solution pH values of 1 to 11, showing an excellent adaptability to solution pH. The removal efficiency increased with the steel slag dosage and a decrease in the initial vanadium concentration. The optimum conditions for vanadium removal were steel slag dosage of 50 g/L and particle size <0.15 mm, and the removal efficiency reached 97.1% for an initial vanadium concentration of 100 mg/L. The adsorption followed the Freundlich model. The washing reagent residue greatly influenced the vanadium removal. Citric acid, tartaric acid, and Na_2_EDTA all caused obvious decreases in the removal efficiency. In contrast, when the oxalic acid concentration was less than 0.2 mol/L, the vanadium removal efficiency remained at 98%, and when the oxalic acid concentration exceeded 0.2 mol/L, the removal rate decreased. Therefore, oxalic acid of 0.2 mol/L is recommended as the washing reagent for vanadium contaminated soil, because it can remove vanadium from soil effectively and make it easier to deal with the washing effluent.

### Equipment and settings

All the figures in this manuscript were made using Origin 9. The two parts of Figs 3 and 4 were arranged as a single file using Photoshop. These figures were adjusted to 300 dpi resolution using Photoshop. We have not conducted excessive manipulations.

## Electronic supplementary material

Supplementary information
