# Supplementary material for: BOF steel slag as a low-cost sorbent for vanadium (V) removal from soil washing effluent
Source: Sci Rep. 2017 Sep 11;7:11177. doi: 10.1038/s41598-017-11682-3 (PMC5594007; doi:10.1038/s41598-017-11682-3)
Supplement: Supplementary file 1 — Supplementary information [file 41598_2017_11682_MOESM1_ESM.doc]

**Supplementary information**

**for**

**BOF steel slag as a low-cost sorbent for vanadium (V) removal from soil washing effluent**

**Authors:** Yuchen Gao, Jianguo Jiang, Sicong Tian, Kaimin Li, Feng Yan, Nuo Liu,

Meng Yang, Xuejing Chen


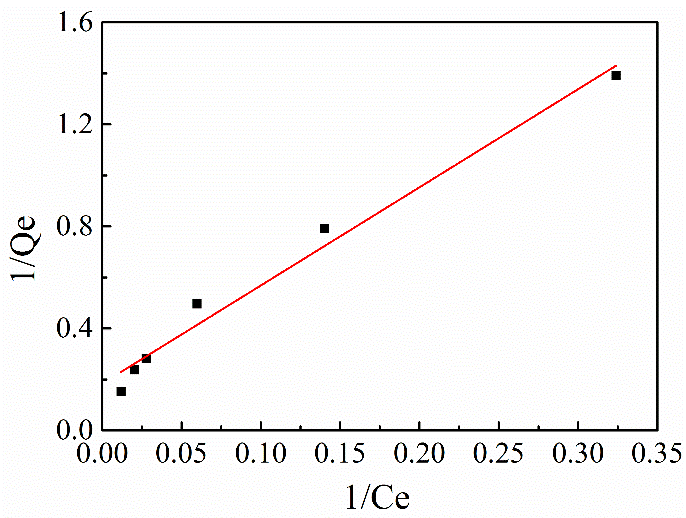
**
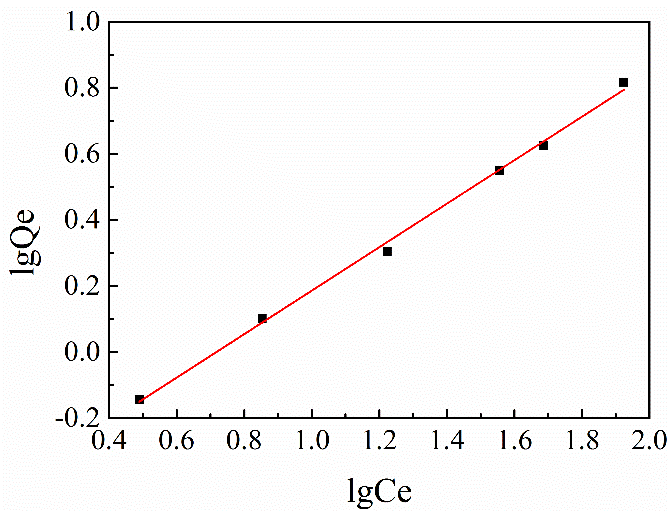
**

1. (b)

**Fig. S1.** Linear fit for (a)Langmuir model, (b)Freundlich model.

| 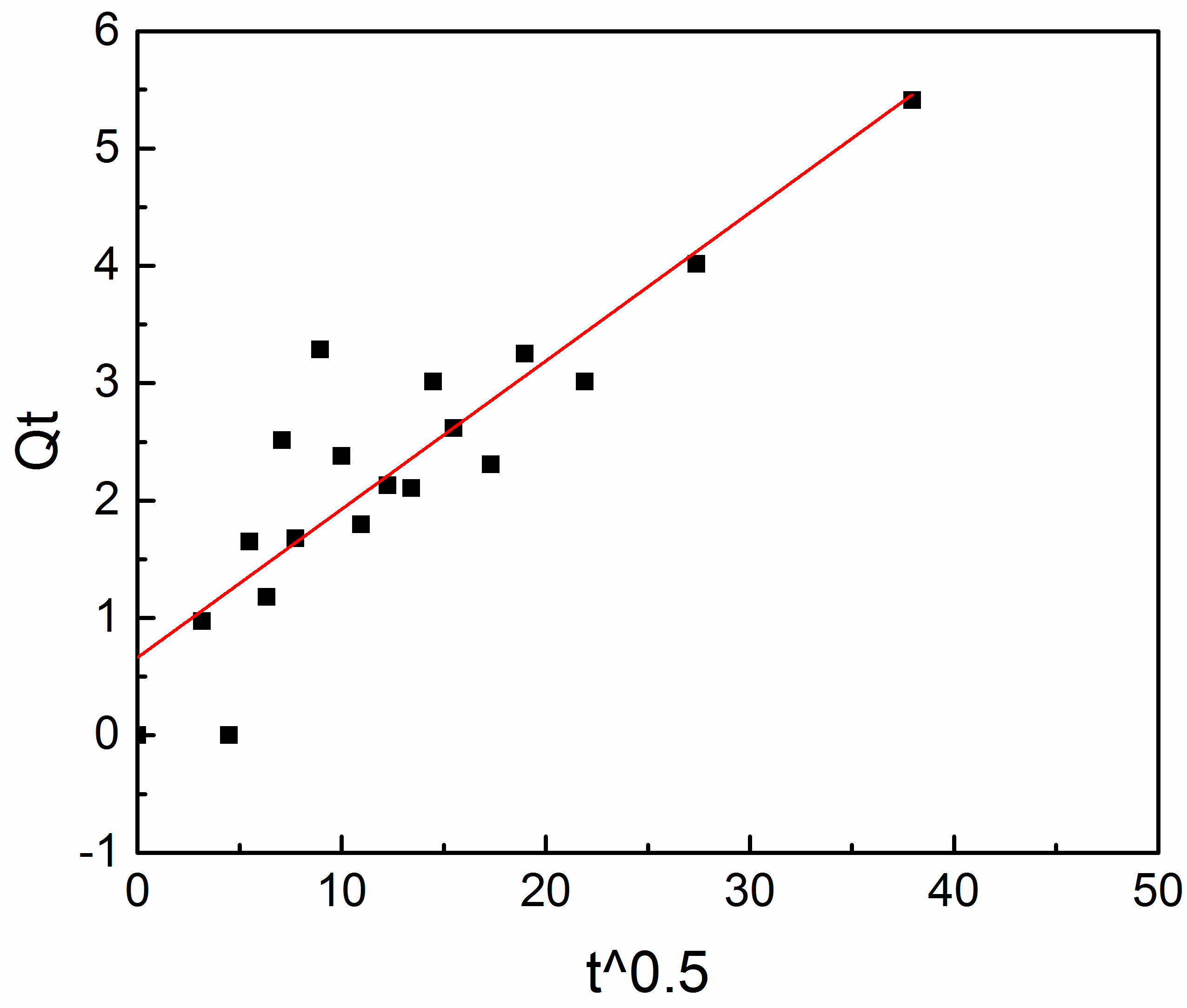  (a) | **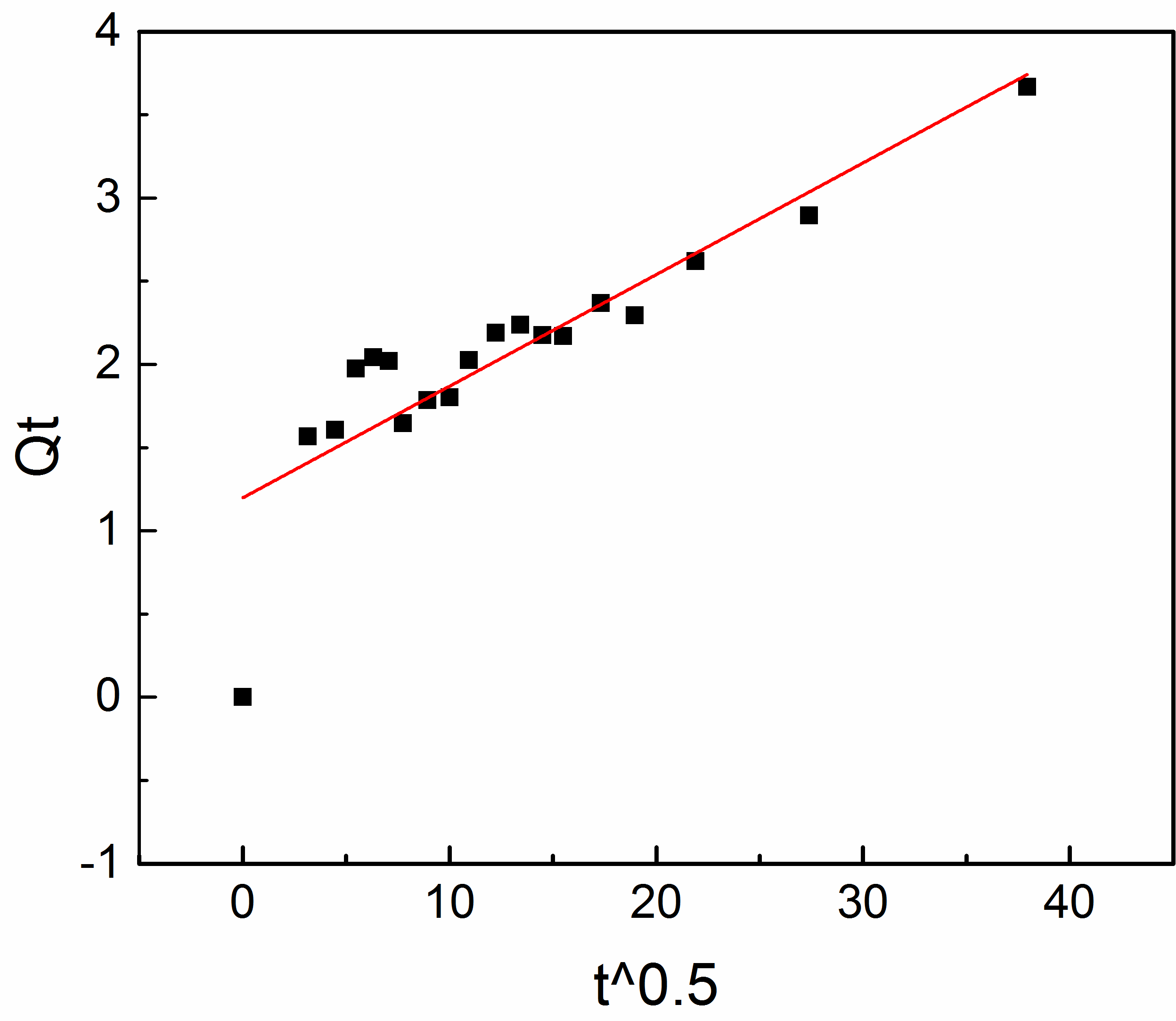**  (b) |
| --- | --- |
| 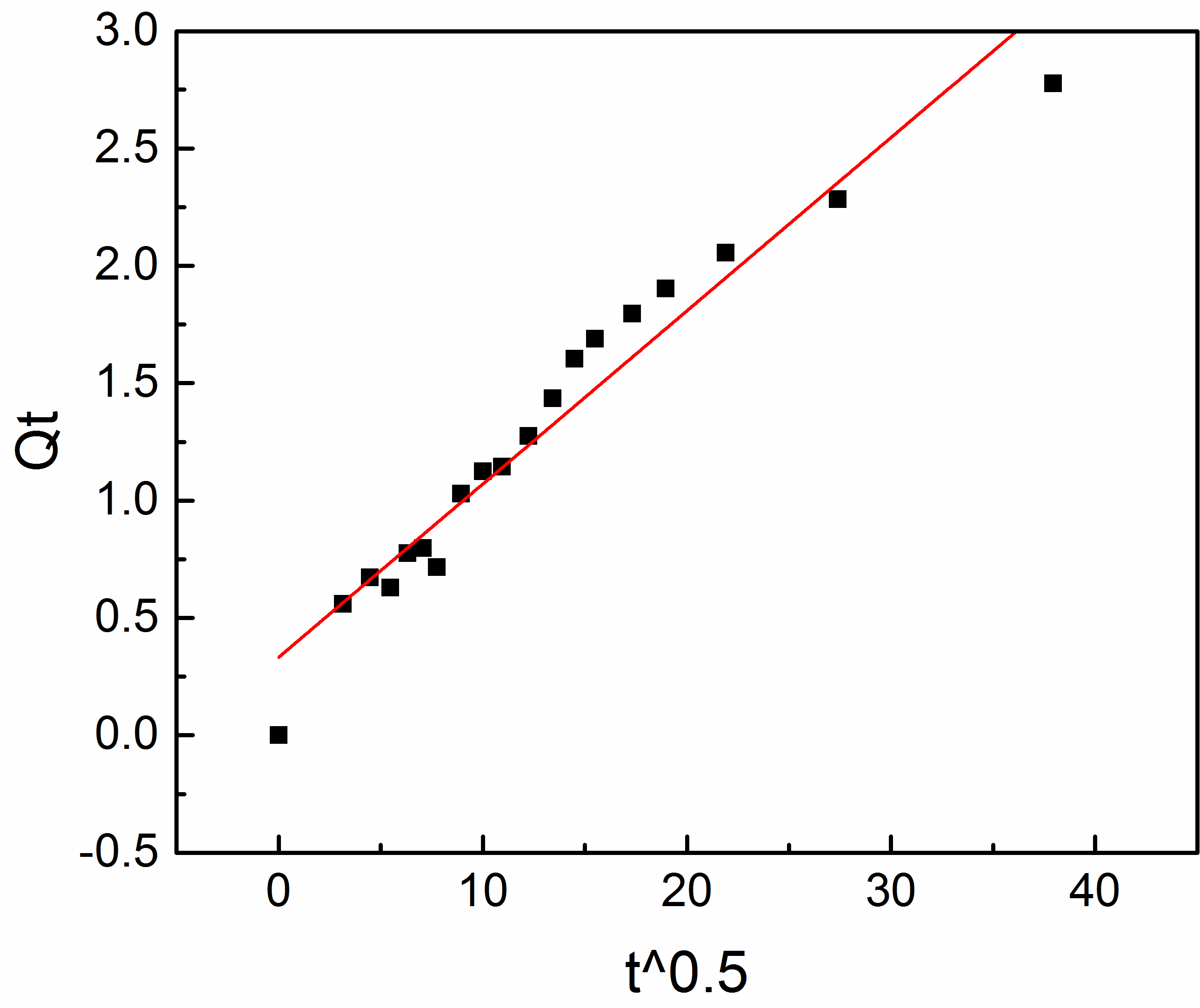  (c) | 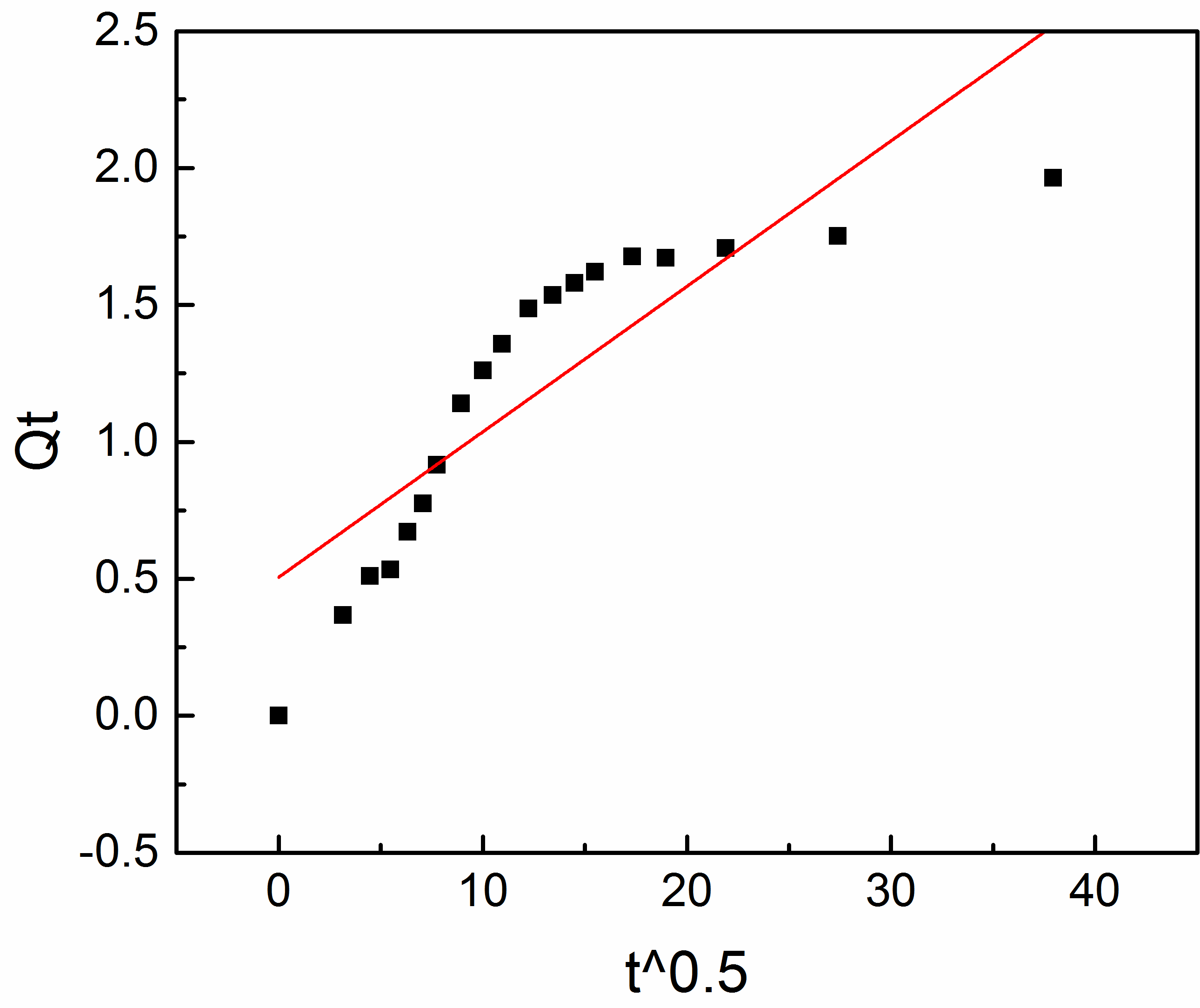  (d) |

**Fig. S2.** Linear fit for intraparticle diffusion model with steel slag dosage (a)0.5g, (b)1.0g, (c)2.5g, (d)5.0.

The integrating equations in linear form of the pseudo-first-order model and pseudo-second-order model are as following:

$\log(Q_{e}-Q_{t})=\log Q_{e}-\frac{k_{1}}{2.303}t$ (S1)

$\frac{t}{Q_{t}}=\frac{1}{k_{2}Q_{e}^{2}}+\frac{t}{Q_{e}}$ (S2)

| 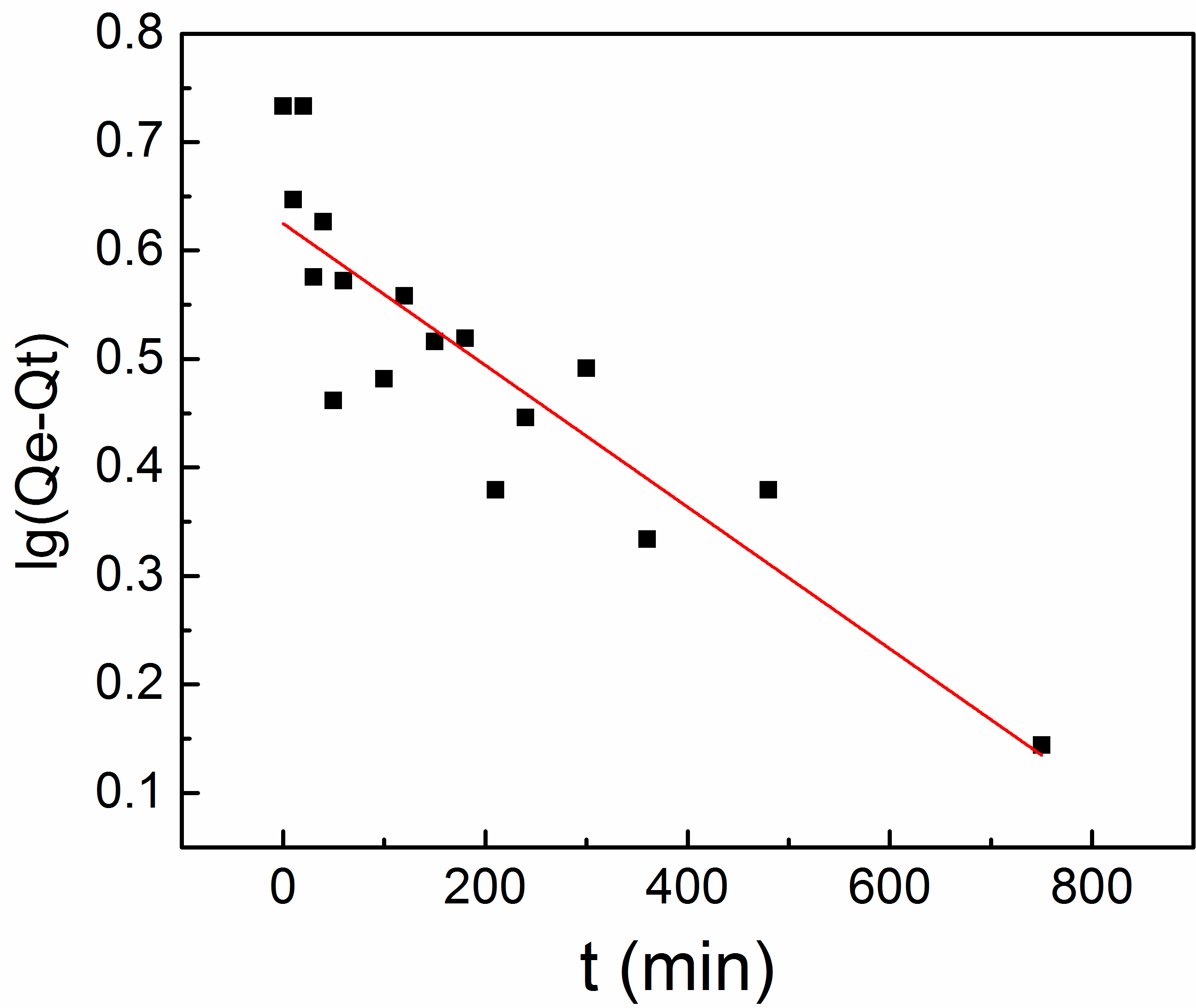  (a) | **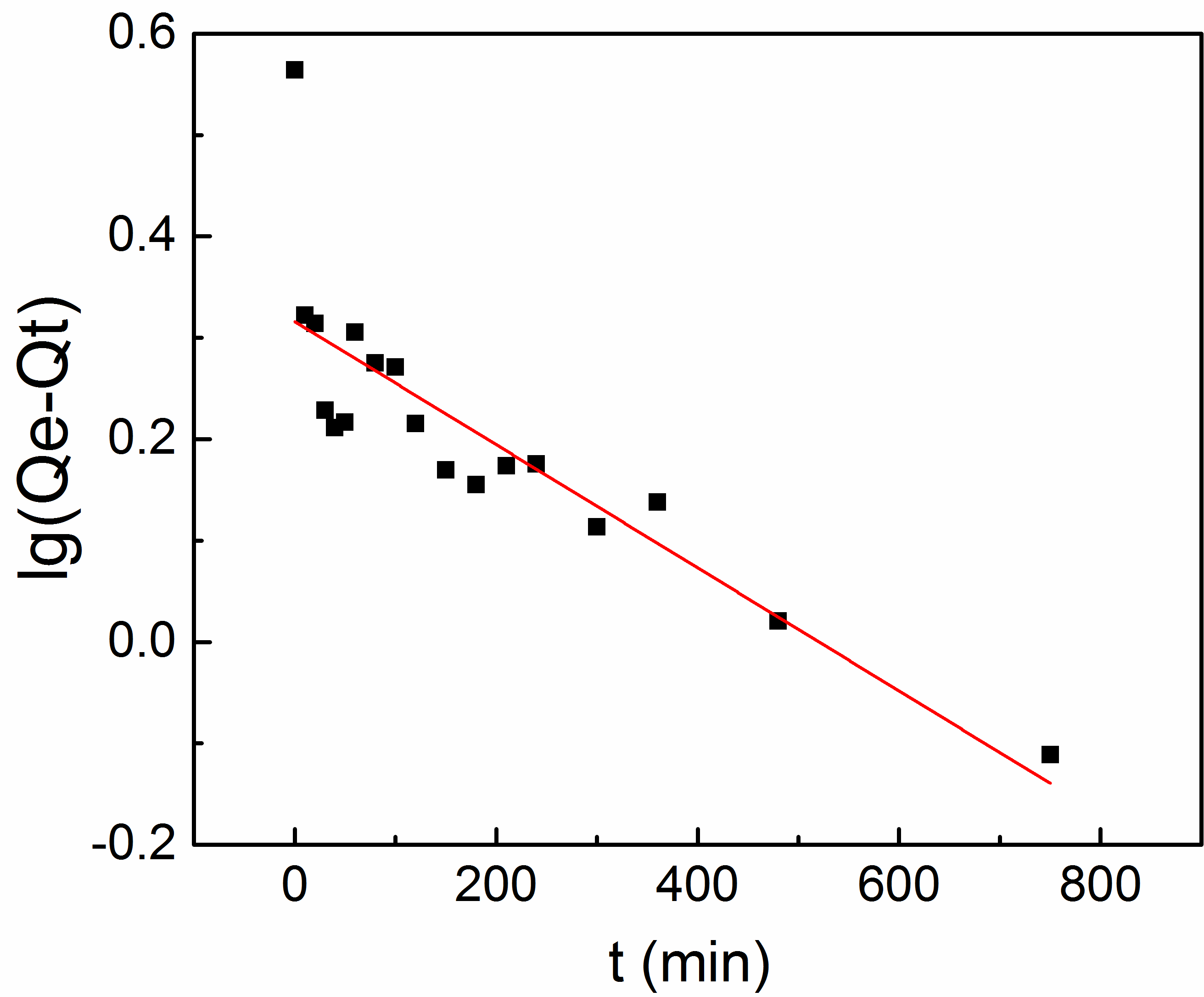**  (b) |
| --- | --- |
| 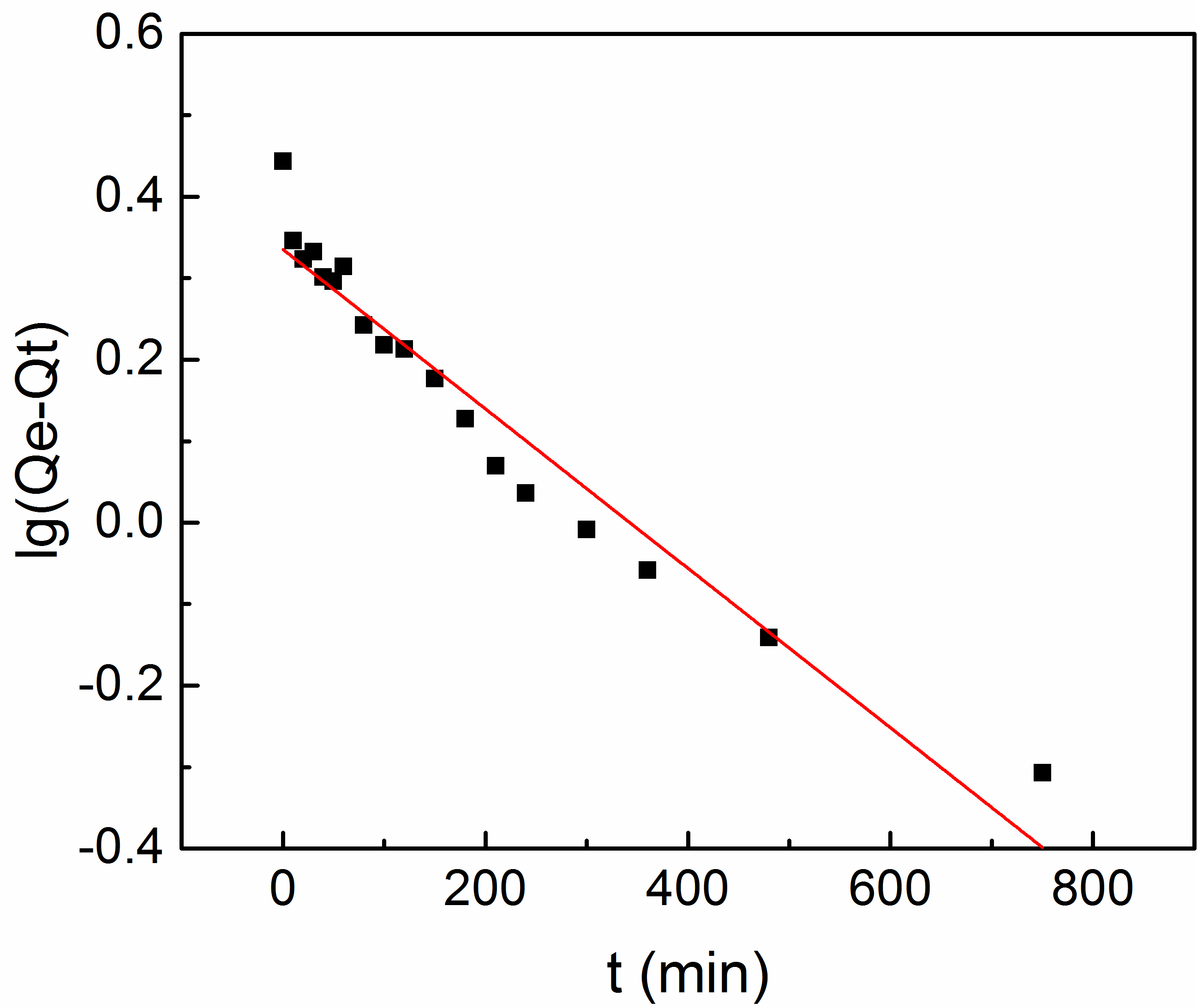  (c) | 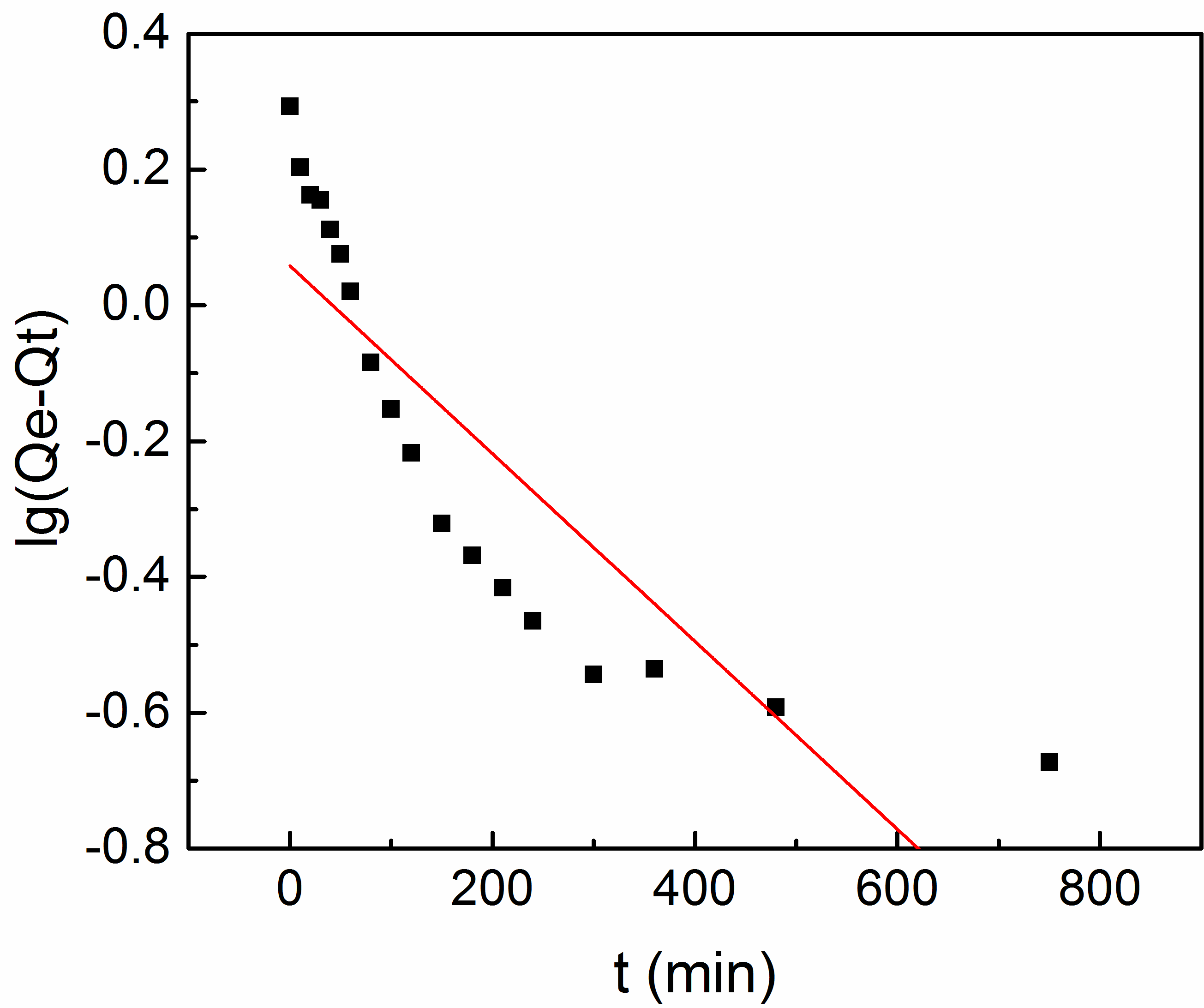  (d) |

**Fig. S3.** Linear fit for pseudo-first-order model with steel slag dosage (a)0.5g, (b)1.0g, (c)2.5g, (d)5.0.

| 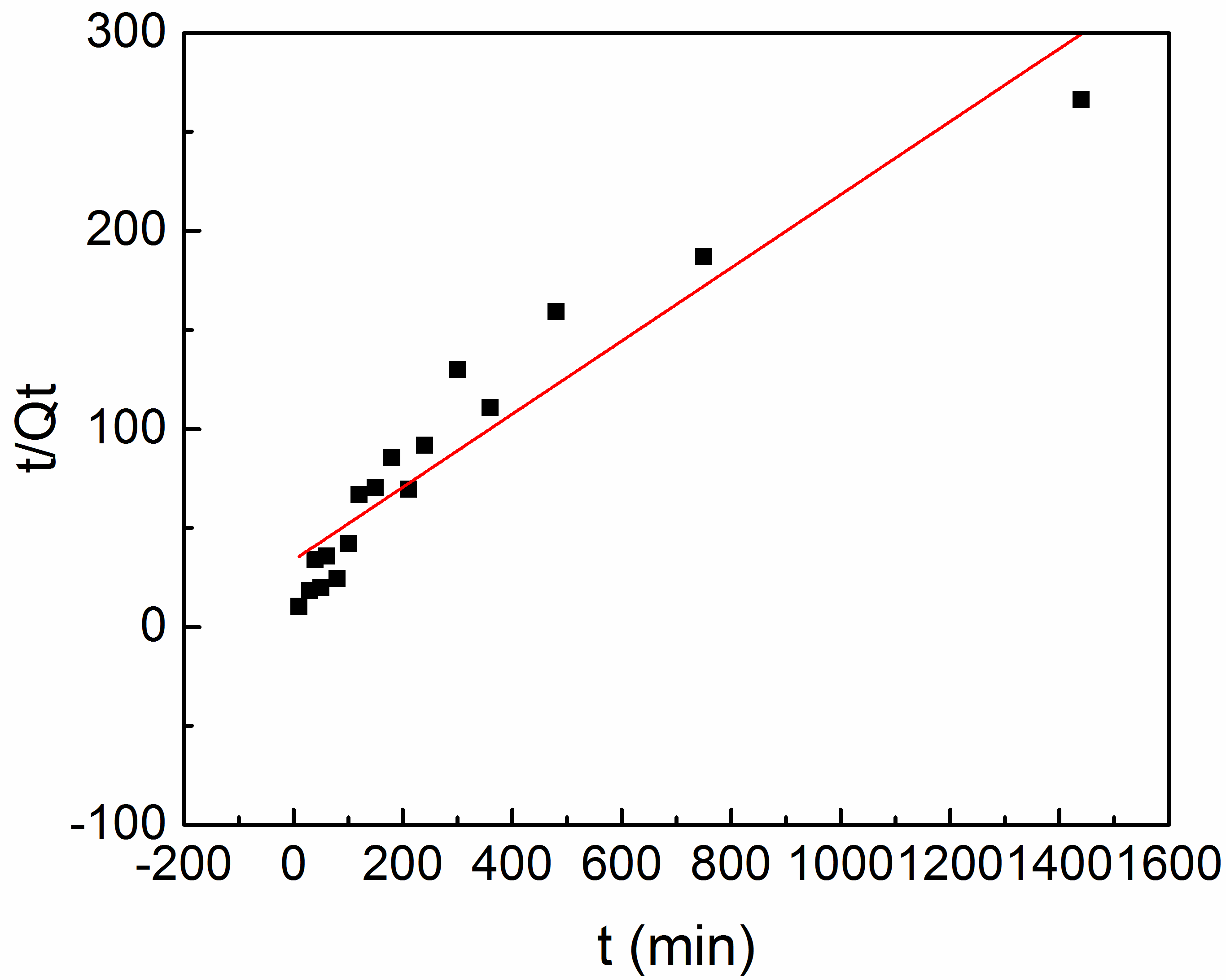  (a) | **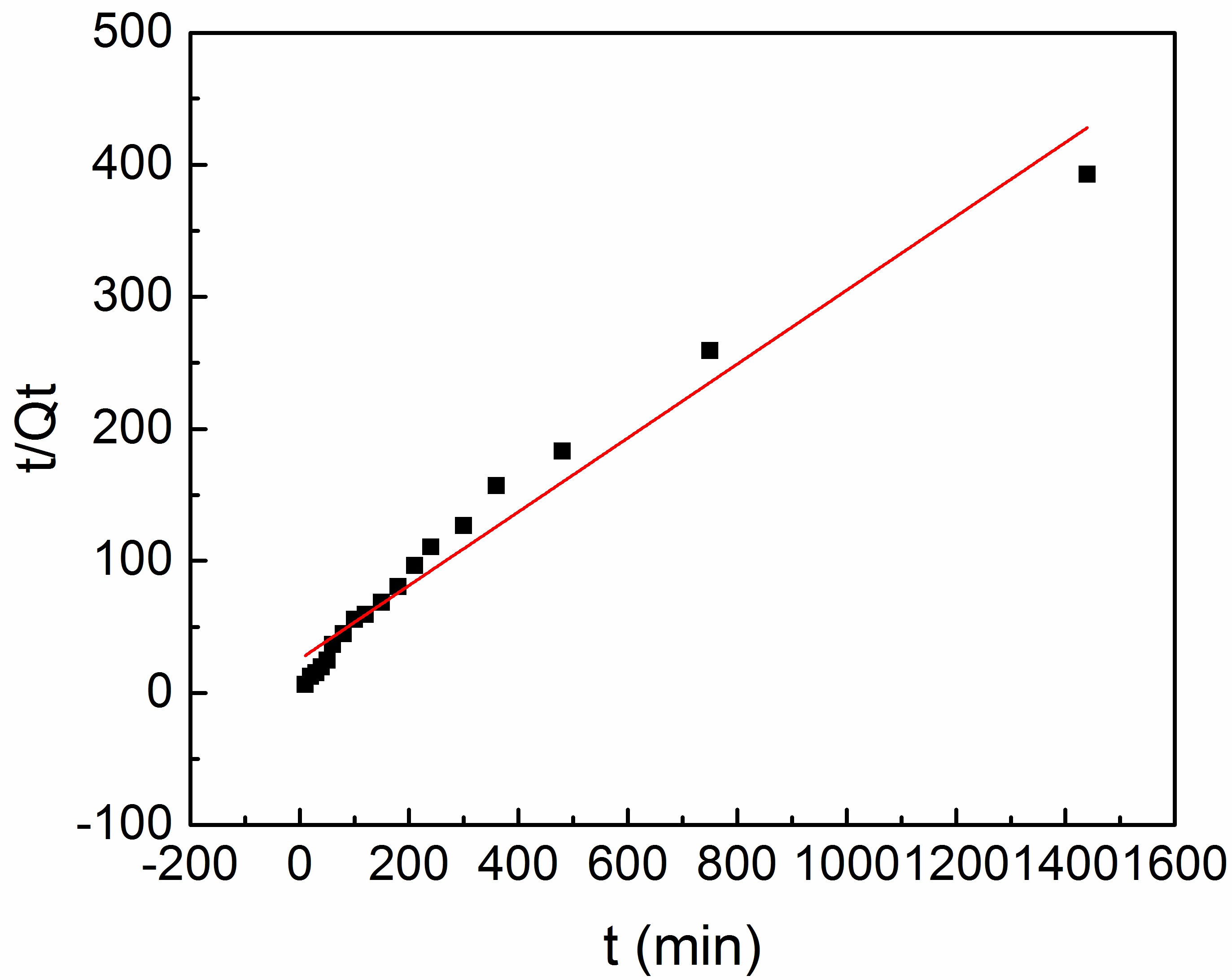**  (b) |
| --- | --- |
| 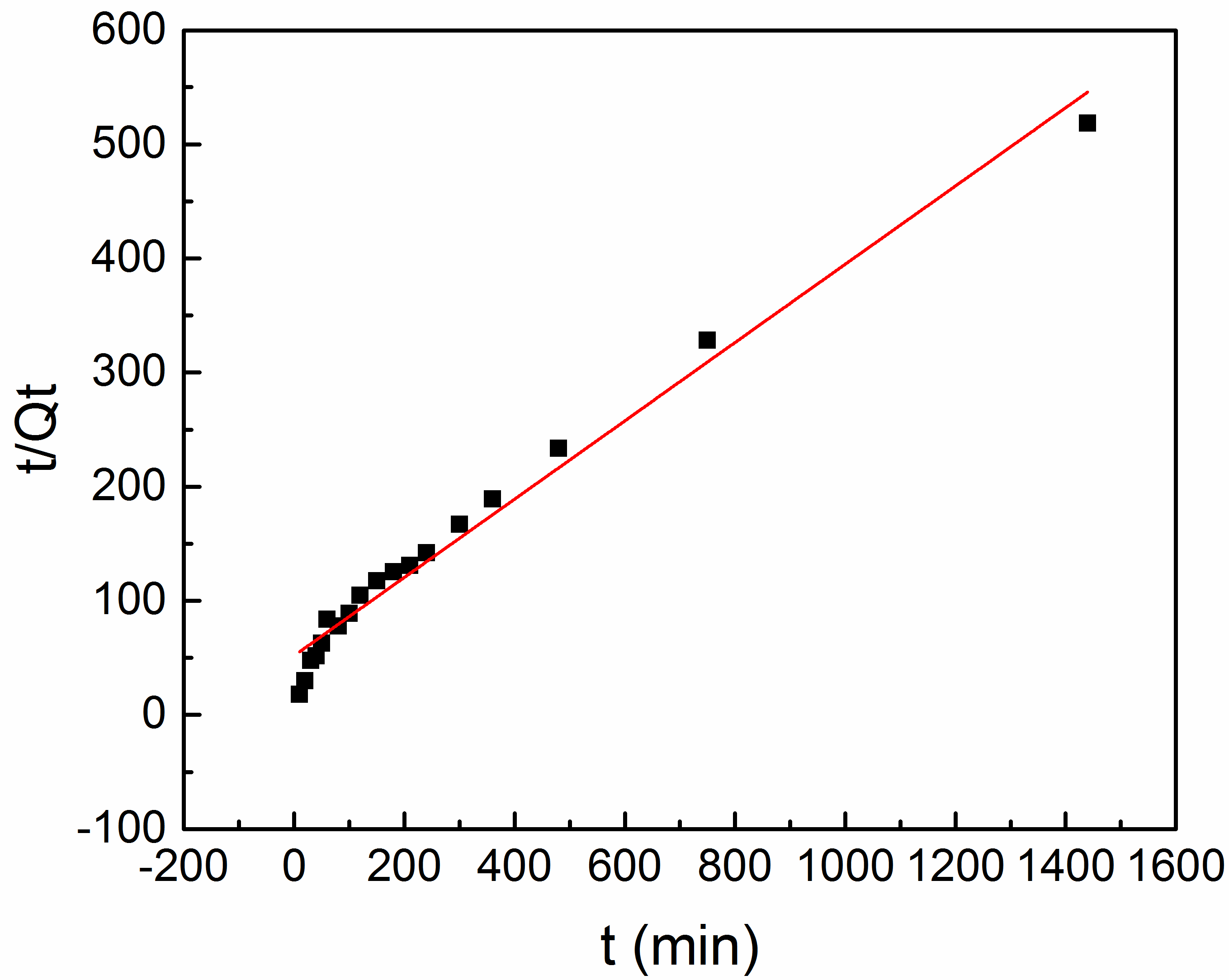  (c) | 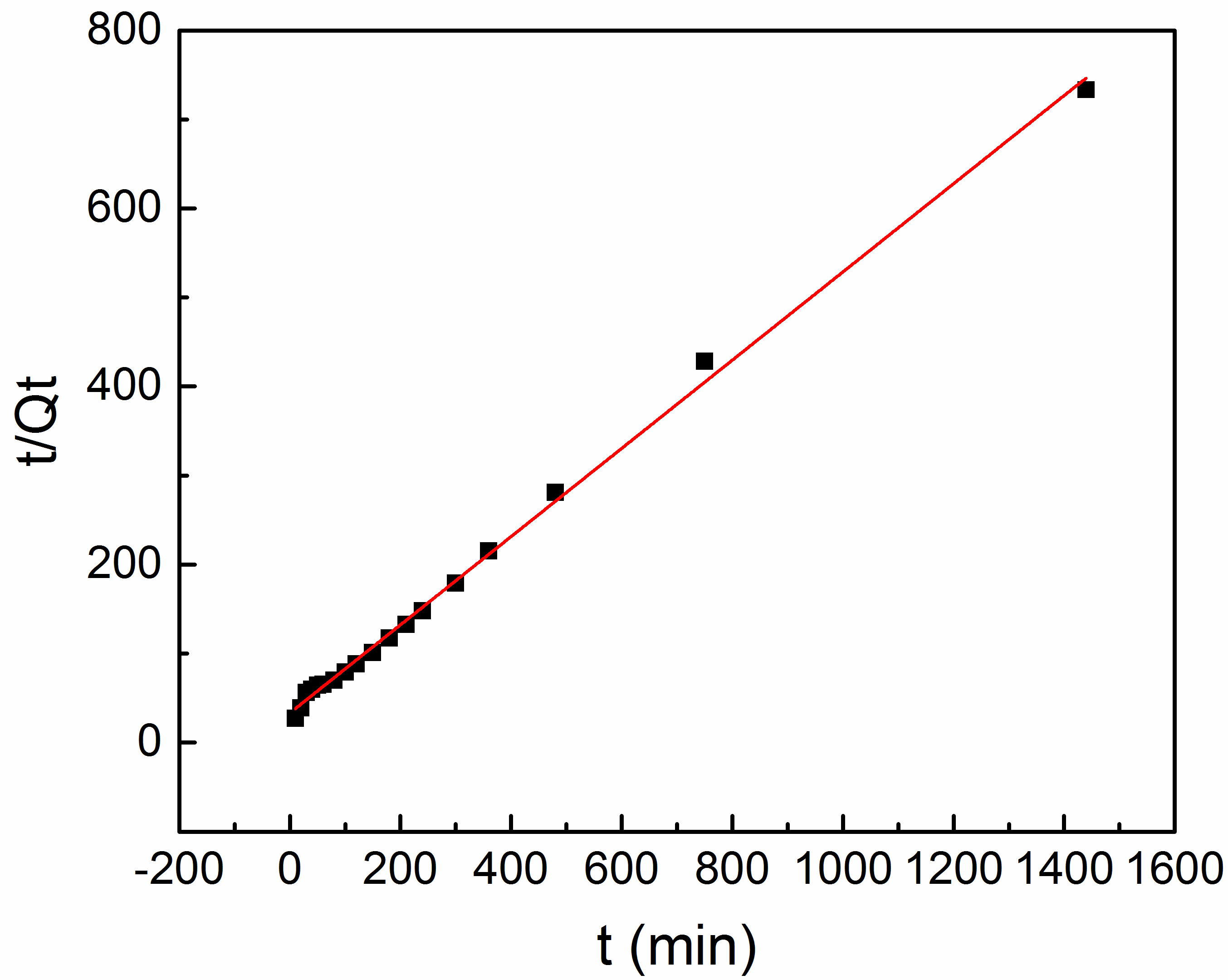  (d) |

**Fig. S4.** Linear fit for pseudo-second-order model with steel slag dosage (a)0.5g, (b)1.0g, (c)2.5g, (d)5.0.

**Table S1.** Kinetic parameters of pseudo-first-order model and pseudo-second-order model.

| Dosage(g) | Pseudo-first-order | | | Pseudo-second-order | | |
| --- | --- | --- | --- | --- | --- | --- |
|  | Q_e_（mg/g） | k_1_（×10^-3^ min^-1^） | R^2^ | Q_e_（mg/g） | k_2_（×10^-3^ g mg^-1^ min^-1^） | R^2^ |
| 0.5 | 4.216 | 1.505 | 0.780 | 5.420 | 1.011 | 0.901 |
| 1.0 | 2.071 | 1.399 | 0.730 | 3.578 | 3.072 | 0.965 |
| 2.5 | 2.164 | 2.253 | 0.944 | 2.916 | 2.268 | 0.979 |
| 5.0 | 1.144 | 3.178 | 0.744 | 2.019 | 7.387 | 0.998 |

**Table S2.** Vanadium concentrations in real washing effluents

| Soil sample | 1 | 1 | 1 | 2 | 2 |
| --- | --- | --- | --- | --- | --- |
| Washing reagents | citric acid, 0.4mol/L | tartaric acid, 0.4mol/L | oxalic acid, 0.4mol/L | oxalic acid, 0.1mol/L | oxalic acid, 0.2mol/L |
| V concentration in effluent (mg/L) | 93.2 | 90.5 | 83.64 | 112.1 | 112.3 |

*Soil sample 1 and 2 are two batches of soil from a vanadium ore contaminated site in Hubei Province.


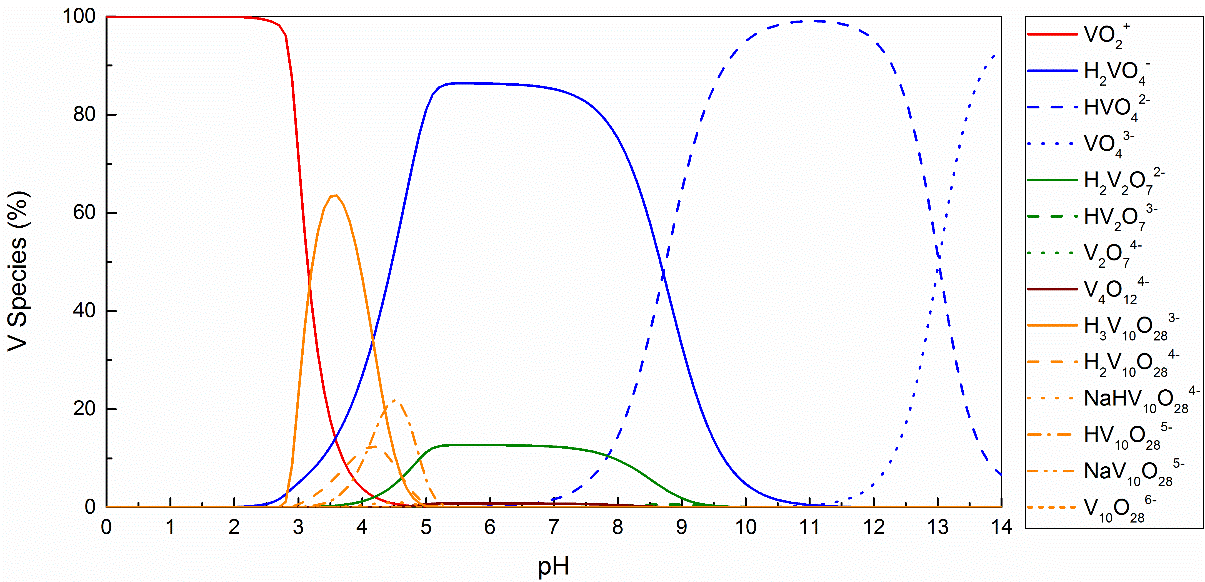


**Fig. S5.** Simulation result of vanadium species in solution under different pH.
